# Delivery mode and perinatal antibiotics influence the predicted metabolic pathways of the gut microbiome

**DOI:** 10.1038/s41598-021-97007-x

**Published:** 2021-09-01

**Authors:** Petri Vänni, Mysore V. Tejesvi, Sofia Ainonen, Marjo Renko, Katja Korpela, Jarmo Salo, Niko Paalanne, Terhi Tapiainen

**Affiliations:** 1 PEDEGO (Pediatrics, Dermatology, Gynecology, Obstetrics) Research Unit and Medical Research Center Oulu, University of Oulu, P.O. Box 5000, 90014 Oulu, Finland; 2 Ecology and Genetics, Faculty of Science, University of Oulu, Oulu, Finland; 3 Department of Paediatrics, University of Eastern Finland and Kuopio University Hospital, Kuopio, Finland; 4 Department of Pediatrics and Adolescent Medicine, Oulu University Hospital, Oulu, Finland; 5 Biocenter Oulu, University of Oulu, Oulu, Finland

**Keywords:** Paediatric research, Clinical microbiology

## Abstract

Delivery mode and perinatal antibiotics influence gut microbiome composition in children. Most microbiome studies have used the sequencing of the bacterial 16S marker gene but have not reported the metabolic function of the gut microbiome, which may mediate biological effects on the host. Here, we used the PICRUSt2 bioinformatics tool to predict the functional profiles of the gut microbiome based on 16S sequencing in two child cohorts. Both Caesarean section and perinatal antibiotics markedly influenced the functional profiles of the gut microbiome at the age of 1 year. In machine learning analysis, bacterial fatty acid, phospholipid, and biotin biosynthesis were the most important pathways that differed according to delivery mode. Proteinogenic amino acid biosynthesis, carbohydrate degradation, pyrimidine deoxyribonucleotide and biotin biosynthesis were the most important pathways differing according to antibiotic exposure. Our study shows that both Caesarean section and perinatal antibiotics markedly influence the predicted metabolic profiles of the gut microbiome at the age of 1 year.

## Introduction

The perinatal period plays a critical role in gut microbiome development. Several studies have shown that the gut microbiome composition of infants delivered by Caesarean section (C-section) shows a reduced diversity^1,2^, lower relative abundance of *Bacteroides*^1–5^ and a higher relative abundance of *Enterococcus*^2,3,5^*, Klebsiella*^3,5^ and *Clostridium*^3,4^ as compared to that of vaginally delivered infants^1,3^. Currently, perinatal antibiotics are frequently used during both vaginal deliveries and C-sections to prevent early-onset group B streptococcal (GBS) sepsis and after birth to treat suspected neonatal infections^6^. Intrapartum antibiotic prophylaxis (IAP), administered to mothers during delivery to prevent GBS transmission to newborn infants, has been associated with reduced gut microbiome richness^7^ and diversity^8–10^, lower relative abundance of Bacteroidetes^7,8,11^ and *Bacteroides*^3,7,10,11^ and a higher relative abundance of Proteobacteria^8,9^ and Firmicutes^9,11^ in term vaginally delivered infants.

The human gut microbiome is highly functionally redundant and different taxonomic compositions can share similar metabolic functions^12,13^. In most earlier paediatric cohort studies^2,4,5, 7–9,11^, gut microbiome composition has been presented based on the sequencing of bacterial 16S gene, commonly used as a marker gene in microbiome studies. Yet, the metabolic function of the gut microbiome has seldom been investigated in paediatric cohorts. Whole genome sequencing (WGS) of all bacteria in gut microbiome would be of great benefit, as WGS identifies the taxa and gene composition with a higher resolution compared to 16S method^14^. WGS, however, has rarely been used for large datasets because it is expensive and laborious. In recent years, multiple advanced bioinformatics tools have been developed to overcome these problems, such as PICRUSt2^15^, Tax4Fun2^16^ and Piphillin^17^. These tools predict the functional pathways of the microbiome based on 16S rRNA sequences and produce results resembling bacterial whole genome sequencing metabolic pathway data.

Our hypothesis was that early perinatal events may markedly change the metabolic pathways of the gut microbiome and influence the later health of children. In the present study, we characterized and compared the effects of delivery mode and perinatal antimicrobial exposure on the predicted metabolic pathways of the gut microbiome in infants at 1 year of age.

## Methods

### Study design and population

We used predicted functional profiles of gut microbiomes based on 16S data from two prospective cohorts from our laboratory.

The delivery mode cohort (DM cohort) consisted of 212 consecutive newborn infants born at the Central Finland Central Hospital in Jyväskylä, Finland, between February 2014 and March 2014, recruited in the delivery room. The patients of the cohort were term or near-term infants. For the analysis, they were grouped into two groups based on the mode of delivery: (1) vaginally delivered (N = 60) and (2) born by C-section (N = 23). Reported sample sizes above were the number of samples that did not get removed in various pre-processing phases. The detailed information on the study population and sample collection has been reported previously^18^. The Ethics Committee of the Central Finland Hospital District found the study plan ethically acceptable (decision number 1E/2014).

The perinatal antibiotic cohort (PA Cohort) consisted of 149 vaginally delivered term infants born at the Oulu University Hospital in Oulu, Finland, between February 2014 and June 2015. The participants were recruited the first day after birth. The participants were recruited according to their perinatal antibiotic exposure and classified into two groups: (1) the control group and (2) those with any perinatal antibiotic exposure. In the control group (N = 27), the infants were not exposed to any perinatal antibiotics. In the antibiotic exposure group (N = 70), either the mother received antibiotics during delivery, the infant received antibiotics during the first days of life, or both received antibiotics. Sample sizes above were the number of samples that were not removed in pre-processing phases. The background characteristics and antibiotic exposures of the participants have been previously reported in detail^11,19^. The research plan was reviewed and approved by the Regional Ethics Committee of the Northern Ostrobothnia Hospital District, Oulu University Hospital, Oulu, Finland (decision number EETTMK 76/2013).

Parents or caregivers of children in both study cohorts gave written informed consent before the study. The study was conducted in accordance with the relevant guidelines, regulations and legislation regarding clinical studies and data protection. In both cohorts, at the age of 1 year, families collected faecal samples from the infant’s potty or diaper and sent them to the laboratory. The faecal samples were processed similarly and were frozen at temperatures lower than − 22 ℃. All samples were analysed with a similar methodology by 16S rRNA gene sequencing at the University of Oulu, Finland. Details about DNA extraction, primers, and sequencing protocol has been previously published for DM^11^ and PA^19^ data. All the raw sequences were submitted to the GenBank Sequence Read Archive (SRA) with accession numbers SRP152384 and PRJNA605735.

We have previously published the results concerning impact of antibiotic exposure on the gut microbiome 16S composition from birth to the age of 6 months^11^, and compared the impact or oral antibiotic courses and perinatal antibiotics on gut microbiome 16S composition at 12 months^19^ in the PA cohort. The impact of delivery mode on the gut microbiome 16S composition at 12 months has not earlier been reported in DM cohort. Predicted metabolic pathway data, presented in this manuscript, have not earlier been published or submitted for either cohort.

Because microbiome data are high-dimensional, complex, noisy and compositional in nature^20,21^, increasing the false discovery rate of conventional hypothesis testing methods^22^, we used a machine learning (ML) approach in this analysis^21,23,24^. Sequence pre-processing and strict quality filtering settings in this study were designed to decrease data dimensionality and sparsity. These upstream choices increase machine learning model performance and interpretability, and as such, differ from previously published work for perinatal antibiotics^11,19^. As a result, features that were rare and found in low prevalence were filtered out. We now present side-by-side results from ANCOM2, ALDEx2, and beta diversity for both cohorts to increase the interpretability of the machine learning models, and effectively showcase the differences in the PA and DM results.

### Sequence pre-processing

Raw sequences from both datasets were imported into Qiime2^25^ (version 2019.10), where they were processed independently from each other. Sequencing primers were trimmed before denoising with the q2-dada2 -plugin. Reads shorter than 270 bp were truncated in the PA cohort and reads shorter than 385 bp were truncated in the DM cohort with an additional 15 bp trimmed from the left side during DADA2^26^. Any quality filtering was avoided before using DADA2.

Taxonomic classifiers were trained using the 132 SILVA^27^ database trimmed to the study primers and truncated using the DADA2 parameter values for truncation and filtering. DADA2 outputs an ASV-table (Amplicon Sequence Variant), which represents the abundances of biological features found from the raw sequences. Features from the ASV-tables were assigned taxonomies and those features were then classified into domains. Features classified as Bacteria and those found in more than one sample were kept. Chimeric features were removed with the q2-vsearch-plugin using the uchime-denovo tool. Samples with a combined feature frequency of less than 1000 were removed. The PA cohort had 1037 minimum depths, while the DM cohort had 1290. We chose 1000 as the depth because it was near to the minimum and fit our analysis methods. Unstratified MetaCyc^28^ pathway abundances were predicted using the q2-picrust2-plugin using the “mp” hidden-state prediction method with other parameters set to default values. Stratified results, which map each predicted pathway back to the input ASV’s, were produced using the original python implementation of PICRUSt2^15^ with the same parameters. ASV and metabolic pathway feature tables from both cohorts were independently analysed.

### Alpha and beta diversity

ASV-tables and predicted pathway tables were used in both alpha and beta diversity analyses in both cohorts. Bray–Curtis dissimilarity was chosen for both types of feature tables for beta diversity, while Shannon index was chosen for alpha diversity. Feature tables were rarefied to depths of 1000 and 10,000 in ASV-tables and predicted pathway tables, respectively. Principal coordinates analysis (PCoA) was done for Bray–Curtis dissimilarity distance matrices. Alpha and beta diversity analyses were done using the q2-diversity-plugin and visualized with Matplotlib^29^.

The Kruskal–Wallis H test was performed to test within-sample group differences for alpha diversity. Adonis PERMANOVA analyses were done using the pairwise beta-diversity-significance command in q2-diversity to test group differences. Adonis PERMANOVA was used for multivariate analysis using both perinatal antibiotic exposure and delivery mode in DM cohort, while only perinatal antibiotics was used with the PA cohort. PCoA analysis was performed with the beta diversity distance matrix and visualized with ellipses drawn onto the two-dimensional space using the Pearson correlation coefficient of the two principal coordinates with the highest explained variance.

### Differential abundance analysis

ALDEx2^30^ and ANCOM^22^ were used for differential abundance analysis to examine group differences in both genus level and predicted pathway data. Features that were found in 10% or more samples were kept. ALDEx2 analyses were done using the q2-aldex2-plugin with a Q-score significance threshold of 0.05. ANCOM2 (further developed based on ANCOM^22^) analyses were done using the R-package^31^ with default parameters. For DM cohort the ANCOM2 analyses were adjusted according to perinatal antibiotic treatments. ANCOM2 calculates a threshold value for the proportion of feature ratios that show significant differences. Prior to testing, we chose the value of 0.7 or higher as the significant value, meaning that when output was 0.7 or higher, ANCOM2 found the feature to be significantly different between study groups. Values below 0.7 were considered non-significant. A significance threshold of 0.7 was recommended by the author of ANCOM2^31^ as a common choice. ALDEx2 uses p-values from Benjamini–Hochberg adjusted (to control false discovery rate) Welch t-tests (p-value of < 0.05 was considered significant prior to testing). Additionally, ALDEx2 outputs the effect size, which indicates the direction and volume of change of the centred log-ratios. In our study, a positive sign indicated that the feature was more abundant in the C-section or perinatal antibiotics treatment group whereas a negative sign indicated greater abundance in their respective control groups (i.e., vaginal birth and no-perinatal antibiotics treatments).

### Machine learning analysis

Machine learning models were trained to predict the target variables of delivery mode and perinatal antibiotic treatments in DM and PA cohorts, respectively. Models were created using a nested cross-validation (CV) setting where parameters were only tuned using the inner cross-validation loop. Random Forest (RF)^32^, Extremely Randomized Trees (EXTRA)^33^ and Adaptive Boosting (Adaboost)^34^ models were tuned and trained independently of each other. Model performance was tested against the validation fold that was unseen to the models. Performance was evaluated using the Receiver Operating Characteristic (ROC) of the Area-Under-the-Curve (AUC). Model selection was only done in the inner CV folds using the ROC AUC metric. Machine learning analyses were implemented using the scikit-learn package^35^. Rank aggregation analysis was used to highlight the key shared features for Random Forest, ExtraTrees and Adaboost models during model training. The importance of each taxon or pathway feature was recorded and compiled in a rank aggregation analysis.

### Cross-study predictions

We aimed to find out whether similar machine learning models were able to differentiate the impact of both perinatal antibiotics and C-section on the predicted metabolic pathway composition of the gut microbiome using a cross-study prediction between two available cohorts. Machine learning models were trained using prevalence filtered feature tables from both taxon and pathway data. Models were given the task to differentiate positive (C-section and perinatal antibiotics treatment) and negative (vaginal delivery and no antibiotic treatments) classes from each other. In this analysis, we chose a prevalence level such that each feature needed to be found in a percentage of all samples in both cohorts to be included. Prior to model building, we tried prevalence cut-offs in the range of 10–50% in both taxa and pathway data. In previous studies, researchers have experimented with prevalence thresholds varying from 1 to 10% in one study^36^, while selecting as high as 45% in another^37^. We chose as high prevalence cut-offs as possible while leaving as many features as possible in both types of data. For metabolic pathway data, the percentage chosen for prevalence cut-off was 50% (240 features) and for taxa data, 30% (12 features). Prevalence thresholds and other data filtering choices were not readjusted after initial model building to prevent leaking information between training and testing folds. RF, EXTRA and Adaboost models were trained using only one cohort’s data using the same scheme described earlier. After each iteration of the nested cross-validation loop, the best RF, EXTRA and Adaboost models were combined into an ensemble classifier. This classifier was then used to predict a randomly sampled subset from the other cohort that was completely unseen by the models. Feature importances were estimated using the prefitted ensemble classifier with permutation_importance function from scikit-learn^35^. Next, we examined if models trained on the PA cohort were biased towards samples that had been exposed to perinatal antibiotics in the DM cohort. We pooled together all the test predictions from C-section and vaginal samples into two groups. Samples that had been exposed to perinatal antibiotic treatments and those that had not been exposed and produced the pooled AUC of both groups. There were no marked differences in the AUC when predicting DM cohort samples that had been exposed to perinatal antibiotics (0.71 AUC) to those that had not been (0.7 AUC).

## Results

### Within and between-sample diversity according to delivery mode and perinatal antibiotics

**Figure 1:**
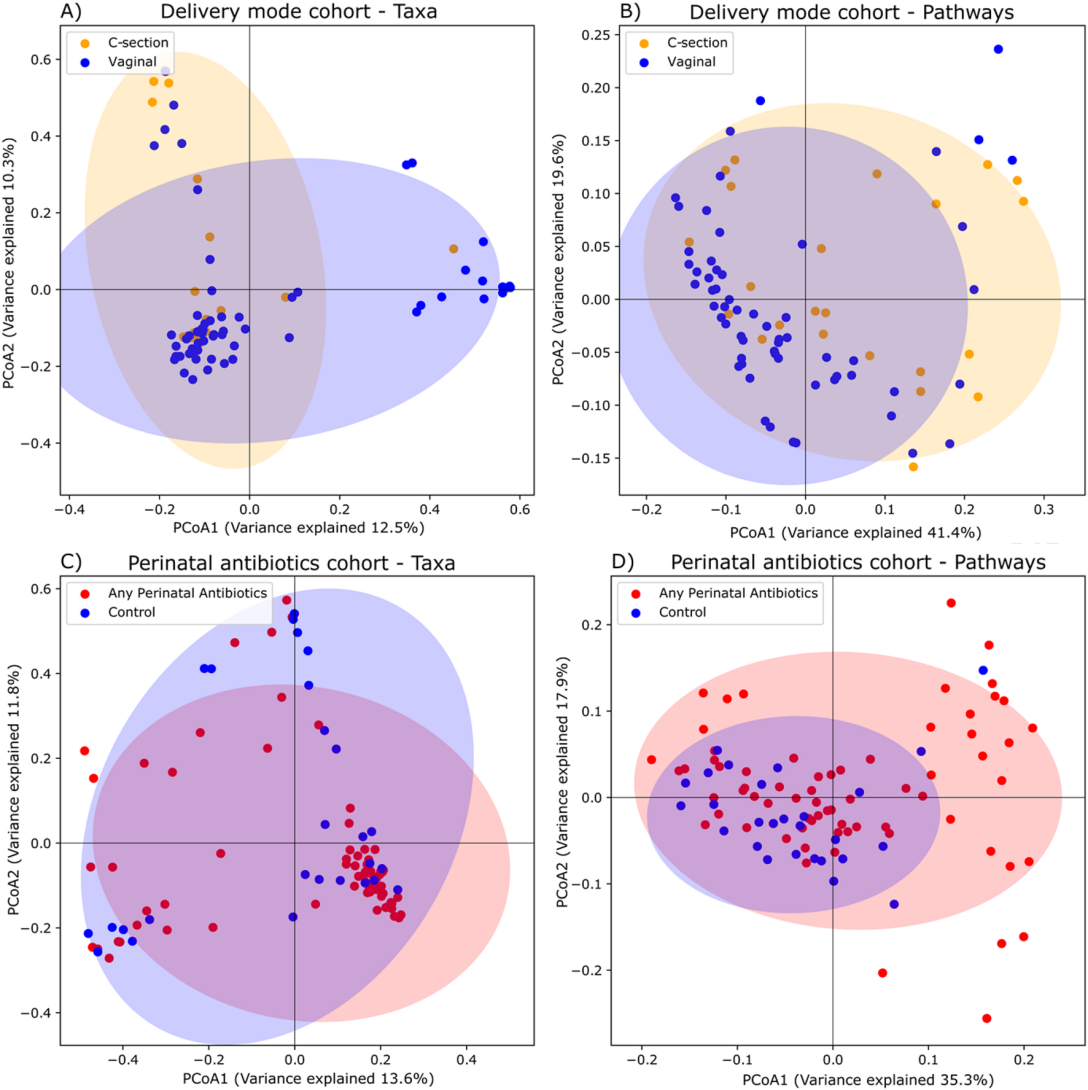
Principal coordinate analysis with Bray-Curtis dissimilarity with taxa and metabolic pathways. Confidence ellipses were drawn based on the Pearson correlation coefficient of the data points. (**A**) Impact of delivery mode on taxonomic composition of gut microbiome (PERMANOVA, *R2* = 0.015, *Pr*(> *F*) = 0.063). (**B**) Impact of delivery mode on metabolic pathway data composition (PERMANOVA, *R2* = 0.017, *Pr*(> *F*) = 0.3). (**C**) Impact of perinatal antibiotic exposure on taxonomic composition of gut microbiome (PERMANOVA, *R2* = 0.020, *Pr*(> *F*) = 0.008). (**D**) Impact of perinatal antibiotics on metabolic pathway data composition (PERMANOVA, *R2* = 0.015, *Pr*(> *F*) = 0.309).

First we analysed beta diversity, i.e., between-sample diversity, to examine differences in the taxonomic and metabolic pathway composition of the gut microbiome according to delivery mode (Fig. 1A,B, delivery mode cohort) and perinatal antibiotic exposure (Fig. 1C,D, perinatal antibiotics cohort) at the age of 1 year using principal coordinate analysis (PCoA).

Delivery mode was not associated according to PERMANOVA analyses for taxa or predicted metabolic pathways (Supplementary Table 1). Perinatal antibiotics were associated for taxa (*R2* = 0.0196, *Pr*(> *F*) = 0.008), but not for predicted metabolic pathways (Supplementary Table 1).

Alpha diversity metrics, i.e., within-sample diversity, showed no significant differences for taxonomic or metabolic pathway data according to delivery mode or perinatal antibiotic exposure (Supplementary Fig. 1).

### Effects on fatty acid biosynthesis, lipid biosynthesis and biotin metabolism pathways

We then investigated which taxa and metabolic pathways of the gut microbiome were differentially abundant in the gut microbiomes of children depending on the delivery mode (C-section vs vaginal) or perinatal antibiotic exposure (any vs none) (Table 1, Supplementary Table 2). The full output of ANCOM2 and ALDEx2 can be found as “Supplementary Tables S1” (Supplementary Tables 3–10).

**Table 1:** Differential abundance analysis of metabolic pathways in the gut microbiomes of two cohorts using ANCOM2 analysis.

Metabolic pathways	MetaCyc superclass	Perinatal antibiotics (n = 70) vs control (n = 27)	C-section (n = 23) vs vaginal (n = 60)
		Tests passed/n tests	Tests passed/n tests
BIOTIN-BIOSYNTHESIS-PWY*	Biotin metabolism	0.88*	0.99*
GLUCOSE1PMETAB-PWY	Sugar degradation	0.75*	0.01
HISDEG-PWY	Histidine degradation	0.8*	0.01
PWY-5989	Fatty acid biosynthesis	0	0.93*
PWY-6282	Fatty acid biosynthesis	0	0.93*
PWY-6519*	Biotin biosynthesis	0.88*	0.99*
PWY-6572*	Glycosaminoglycan degradation	0.91*	0.97*
PWY-7664	Fatty acid biosynthesis	0	0.91*
PWY0-845	Vitamin B6 metabolism	0.86*	0.65
PWY0-862	Fatty acid biosynthesis	0	0.77*
PWYG-321	Fatty acid biosynthesis	0	0.99*
PYRIDOXSYN-PWY	Vitamin B6 metabolism	0.9*	0.65

Taxon		Perinatal antibiotics (n = 70) vs control (n = 27)	C-section (n = 23) vs vaginal (n = 60)
Bacteroides	–	0.82*	0.90*
Erysipelatoclostridium	–	0.1	0.73*

ANCOM2 outputs a proportion of pairwise statistical tests passed for each feature (tests passed/n tests). For this value, exceeding the threshold of 0.7 was considered significant (bold).

*Proportion > 0.7 for both delivery mode (vaginal delivery vs C-section) and perinatal antibiotic exposure (any vs none) are statistically significant.

Using ANCOM2 analysis for taxa, the abundance of Bacteroides and Erysipelatoclostridium were significantly different in the gut microbiomes of infants born via C-section as compared to that of vaginally born infants (Table 1). When comparing the metabolic pathway data of the gut microbiome, we found several metabolic pathways that were significantly different between functional profiles of gut microbiomes of infants at 1 year of age depending on whether they were born via C-section or vaginal route (Table 1). Five of these pathways were linked to fatty acid and lipid biosynthesis. In ALDEx2 analysis, none of the taxa differed in a statistically significant manner depending on delivery mode, while one pathway related to fatty acid biosynthesis (PWYG-321) was differentially abundant (Supplementary Table 2).

Bacteroides was significantly different depending on exposure to perinatal antibiotics (Table 1). When comparing metabolic pathways according to perinatal antibiotic exposure, we identified seven pathways as significantly different (Table 1), two of which were linked to vitamin B6 metabolism and two to carbohydrate degradation. Three pathways (biotin metabolism, biotin synthesis and glycosaminoglycan degradation) were significantly different according to both the delivery mode and perinatal exposure (Table 1). When we used ALDEx2 for comparisons, we found one genus and 25 differentially abundant metabolic pathways depending on the perinatal antibiotic exposure (Supplementary Table 2).

### Influence of perinatal events on predicted metabolic pathways in gut microbiome

**Figure 2:**
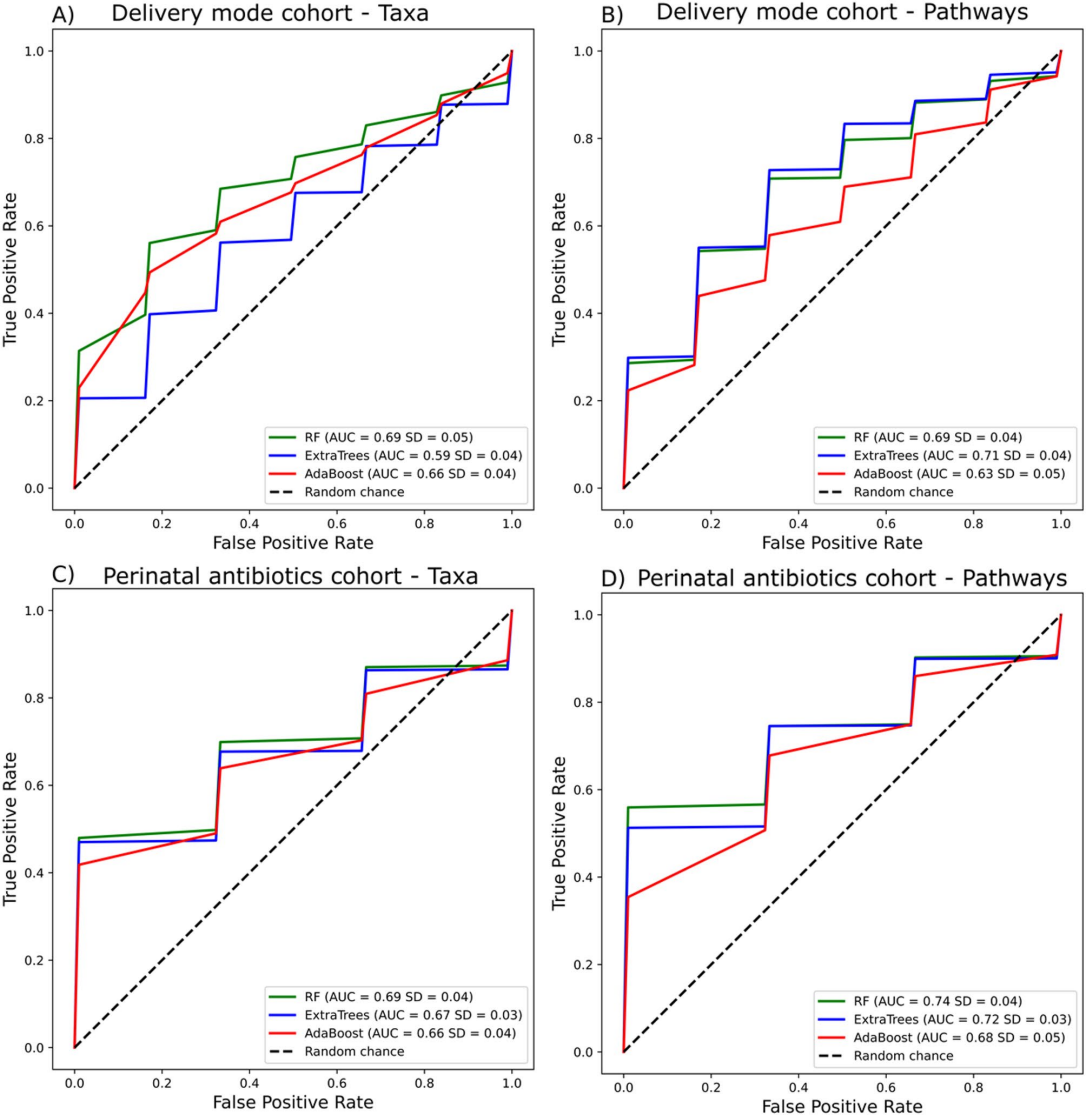
Machine learning model performance when differentiating delivery mode and perinatal antibiotic treatments. Relative abundance data from taxa (**A**, **C**) and metabolic pathways (**B**, **D**) were used. Models can have an area-under-the-curve (AUC) value in the range of 0.5 (random chance) to 1.0 (perfect predictor). The black diagonal line represents random chance performance.

We used machine learning models on predicted metabolic pathways in gut microbiome to differentiate whether the child was born via C-section or vaginal route (Fig. 2A,B), or whether the child was exposed or unexposed to perinatal antibiotics (Fig. 2C,D).

Three algorithms (RF, ExtraTrees, Adaboost) were used to differentiate both target variables using the 16S sequencing derived genera and predicted metabolic pathway data. Models were able to differentiate their respective test samples more precisely when using metabolic pathway data instead of taxa data (Fig. 2).

Children born via C-section were well differentiated from those born vaginally by using machine learning models on gut microbiome data at the age of 12 months (Fig. 2A,B) Using RF for taxa by delivery mode, the highest AUC, representing the measure of separability under ROC, was 0.69 (*SD* = 0.05) (Fig. 2A), while ExtraTrees achieved the highest performance for metabolic pathways of the gut microbiome with an AUC = 0.71 (SD = 0.04) (Fig. 2B), differentiating data by delivery mode.

Similarly, children exposed to perinatal antibiotics were well differentiated from those unexposed by using machine learning models on gut microbiome data at the age of 12 months. RF models trained using taxa to differentiate data by perinatal antibiotic exposure performed better (AUC of 0.69, SD = 0.04) than ExtraTrees or Adaboost (Fig. 2C). Additionally, RF was again the top performer with metabolic pathways in differentiating data by perinatal antibiotic exposure with an AUC of 0.74 (*SD* = 0.04) (Fig. 2D).

### Most important genera and predicted metabolic pathways for machine learning models

**Figure 3:**
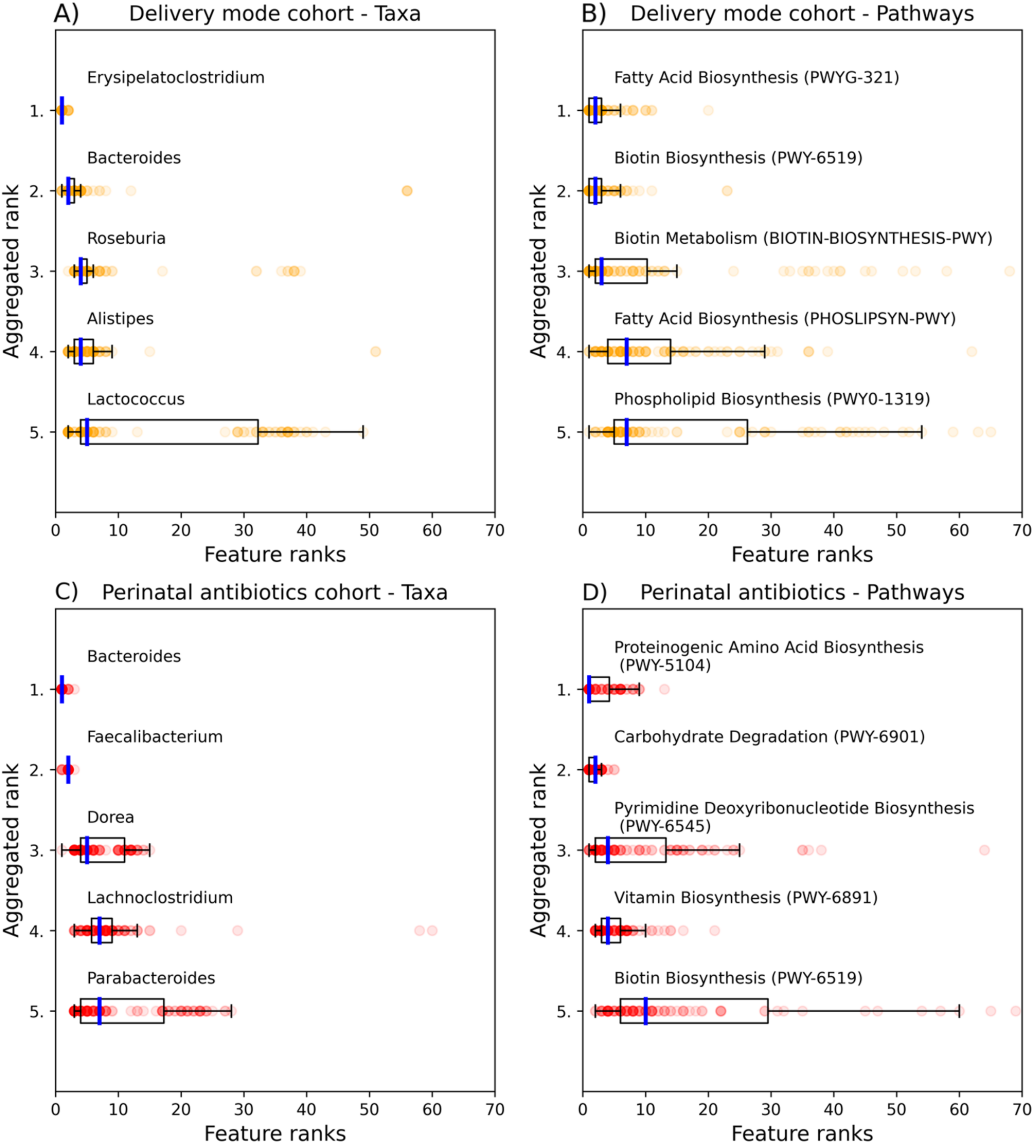
Feature importance rank aggregation analyses. Feature importance values from Random Forest, ExtraTrees and Adaboost machine learning models were transformed into rank values and aggregated together. Data from delivery mode cohort by (**A**) taxa and (**B**) pathways as well as perinatal antibiotics by (**C**) taxa and (**D**) pathways. The analysis was done to determine which features were important among all decision tree-based models. Model training was repeated 40 (red or yellow dots, 120 in total for each feature) times for each algorithm, and the ranks were gathered, and boxplots were drawn using the median. Median ranks were sorted in descending order from most important to the least important. Only the five most important features are shown for visualisation purposes.

Machine learning models are often referred to black boxes, as the inner workings are often obscured or complicated to understand. Random forests and other decision tree-based algorithms have the benefit of providing continuous value for feature importance’s, effectively ranking how well each feature (genera or predicted pathway) affected the overall result. In rank aggregation analysis, the importance of each taxon or pathway feature in machine learning models was recorded and compiled (Fig. 3).

In children born via C-section or vaginally, using gut microbiome data at the age of 1 year, the most important features in gut microbiome 16S composition, differentiating children in machine learning analysis for delivery mode, were Erysipelatoclostridium followed by *Bacteroides*, *Roseburia*, *Alistipes* and *Lactococcus* (Fig. 3A). At the same time, the most important predicted metabolism related pathways, differentiating children in machine learning analysis according to delivery mode, were fatty acid biosynthesis, phospholipid biosynthesis, biotin biosynthesis and biotin metabolism related pathways. (Fig. 3B).

In children exposed or unexposed to perinatal antibiotics, using gut microbiome at the age of 12 months, the most important features differentiating children in machine learning analysis for perinatal antibiotic exposure were *Bacteroides*, *Faecalibacterium* and *Dorea* (Fig. 3C). At the same time, the most important predicted metabolism related pathways, differentiating children in machine learning analysis according to perinatal antibiotic exposure, were proteinogenic amino acid biosynthesis, carbohydrate degradation, pyrimidine deoxyribonucleotide biosynthesis, vitamin biosynthesis and biotin biosynthesis (Fig. 3D).

In both cohorts, there were multiple features with similar median ranks, indicating a high amount of collinearity in pathway data, where many features were correlated with each other. Rank aggregation values for each variable are reported in the “Supplementary File S1” (Supplementary Tables 11–14).

### Cross-study analysis: training models on one cohort and testing them on the other cohort

As the direct comparison of taxon or pathway data from the two datasets using conventional statistics is not optimal, even for datasets from the same laboratory, we then performed a cross-study comparison using machine learning to assess effects associated with delivery mode or perinatal antibiotic exposure (presented in Table 1 and Figs. 2, 3). We examined whether patterns found by machine learning models in one dataset for delivery mode could be generalized to the samples from the other cohort for perinatal antibiotic exposure and vice versa, possibly indicating analogous or similarly differentiating changes in gut microbiome after C-section or perinatal antibiotics as compared to those with undisturbed early gut colonization. We thus trained models on one cohort and tested them on the other cohort.

**Figure 4:**
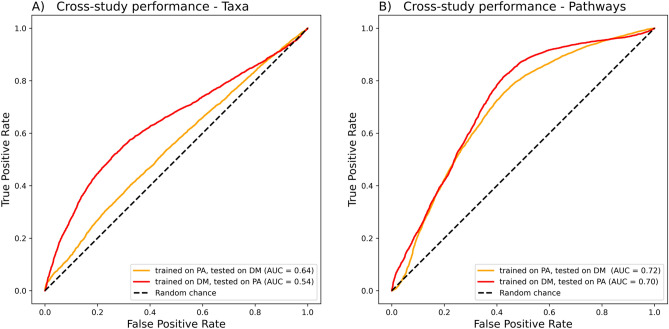
Cross-study performance when machine learning models were trained on one cohort and then tested on the other. (**A**) Taxon and (**B**) metabolic pathway relative abundances were used to train cross-study models. “C-section” and “any perinatal antibiotics” were set as the positive class in their respective cohorts. The yellow line represents performance when models trained on the perinatal antibiotics (PA) cohort was used to predict the samples of the delivery mode (DM) cohort, and the red line represents the reverse situation.

In the cross-study analysis, gut microbiome 16S composition changes due to C-section or perinatal antibiotic were not analogous or similarly differentiating. Model performance was low when using taxon data to train and test models in both cohorts. Models trained on the PA cohort and tested on the DM cohort achieved an AUC of 0.64 (Fig. 4A). Training on the DM cohort and testing on the PA cohort showed an AUC of 0.54.

**Figure 5:**
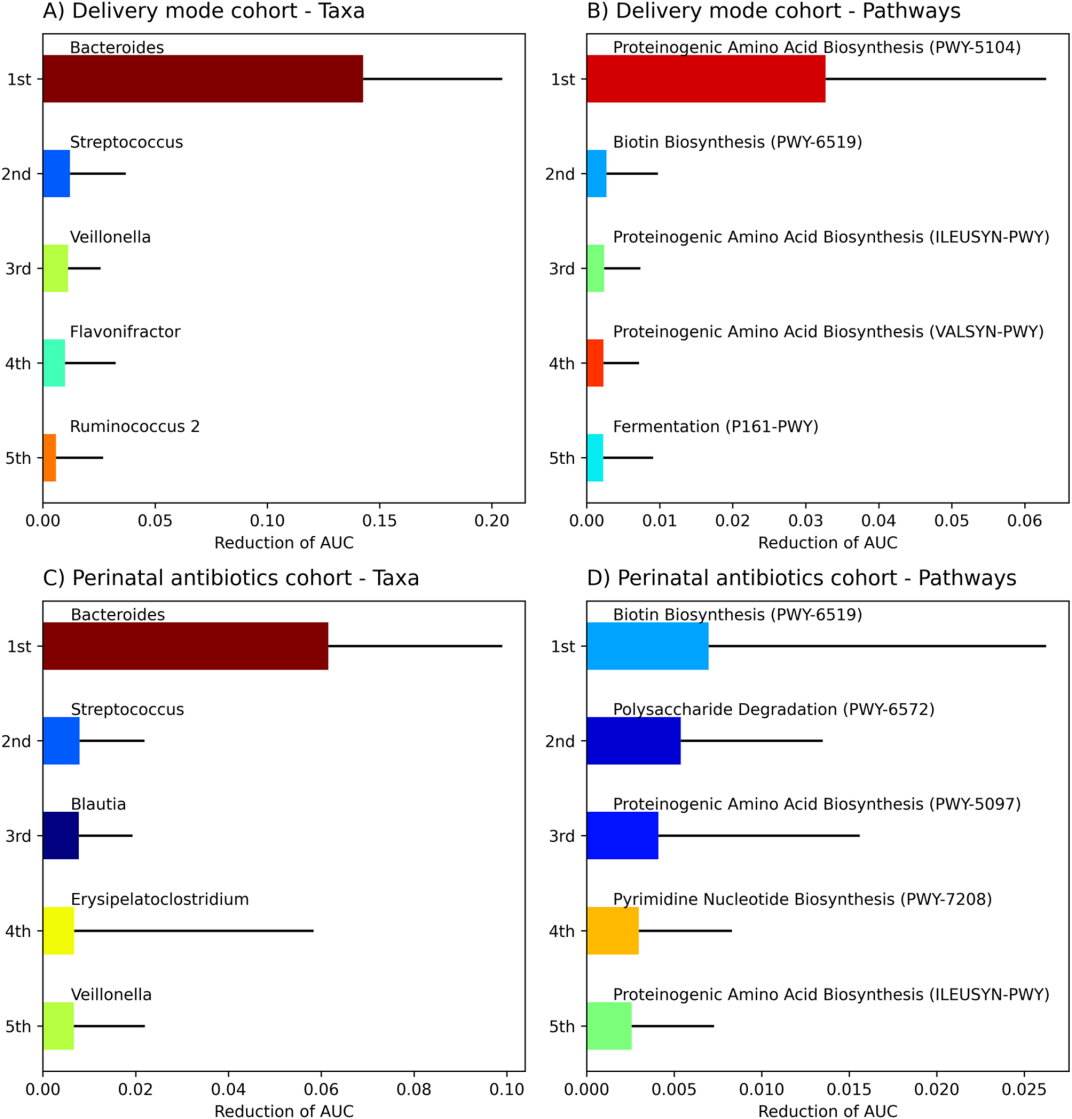
Cross-study importance of taxon and metabolic pathway data for delivery mode (**A** and **B**) and perinatal antibiotic exposure (**C** and **D**). Machine learning models were trained on PA cohort (**A** and **B**) and tested on random DM cohort samples, while models trained using the DM cohort (**C** and **D**) were tested on random PA cohort samples. The sample labels of each taxon or pathway were permutated one at a time in the test samples, and the changes to AUC were recorded and averaged. Positive error bars are plotted for each feature in black.

When using predicted metabolic pathway features, the cross-study models were able to predict cross-study sample classes as positive (C-section or any perinatal antibiotics treatment) or negative (vaginal delivery or no perinatal antibiotics treatments) (Fig. 4B). Models trained on differentiating perinatal antibiotic exposure (any vs none) were able to differentiate the delivery mode (C-section vs vaginal) with an AUC of 0.72, while models trained on delivery mode cohort achieved an AUC of 0.70 in differentiating perinatal antibiotic exposure (Fig. 5B).

### The most important metabolic pathways in the cross-study predictive models

We then analysed which features were most important for the performance of the models by using permutation importance analysis.

After “shuffling”, i.e., deliberately removing the true grouping data for the delivery mode or perinatal antibiotic exposure and replacing it with a random grouping variable, Bacteroides resulted in the largest reduction in the AUC of models in differentiating both delivery modes (mean reduction = 0.14, *SD* = 0.06) (Fig. 5A) and perinatal antibiotic exposure (mean reduction = 0.06, *SD* = 0.04, Fig. 5C) using taxon data from the samples.

After shuffling, using metabolic pathway data, we found several features decreasing only slightly the performance of the model, with the largest average change of 0.03 to AUC for proteinogenic amino acid biosynthesis according to delivery mode (Fig. 5B) and 0.005 for biotin biosynthesis according to perinatal antibiotic exposure (Fig. 5D). In the DM cohort (C-section vs vaginal delivery), pathways related to proteinogenic amino acid biosynthesis, biotin biosynthesis and fermentation were the most important (Fig. 5B). In the PA cohort (any antibiotics vs none in vaginally delivered infants), pathways related to biotin biosynthesis, polysaccharide degradation, proteinogenic amino acid biosynthesis and pyrimidine nucleotide biosynthesis were the most important (Fig. 5D). Values for each tested variable are reported in the “Supplementary File S1” (Supplementary Tables 15–19).

### Relative abundances of important taxa and pathways

Lastly, we present the relative abundances between study groups of genera and pathways that were found significantly different by ANCOM2 (Table 1) or important by machine learning models (Figs. 2 and 4). Additionally, the stratified relative abundance was gathered from PICRUSt2, which allows for a direct link from each predicted pathway back to the ASV’s. Bacteroides was differentially abundant in both DM and PA and was the most important feature of cross-study machine learning models. We additionally stratified the predicted pathway data according to Bacteroides to gain further insight.

In the DM cohort, Bacteroides (Supplementary Table 19) and Erysipelatoclostridium were highlighted by ANCOM2 (Table 1) as differentially abundant, while also being important for machine learning models (Fig. 2A). Bacteroides (36% in C-section and 58% in vaginal) had lower relative abundance in C-section, while Erysipelatoclostridium (6% in C-section and 2.2% in vaginal) was enriched in children exposed to C-section. Several predicted metabolic pathways related to biotin metabolism, biotin biosynthesis, glycosaminoglycan degradation, and fatty acid biosynthesis had decreased relative abundance in C-section children (Supplementary Table 19). Based on Bacteroides stratified data, all the above pathways had 44–99% of their relative abundances come from Bacteroides sequences in C-section samples, while 73–99% of their relative abundances in vaginal group (Supplementary Table 20). One pathway related to fatty acid biosynthesis (PHOSLIPSYN-PWY) and fermentation (P161-PWY) were enriched in C-section samples.

Bacteroides was the only significantly different genera in PA cohort while also being important to machine learning models. Bacteroides had lower relative abundance (38% in perinatal antibiotics and 66% in no antibiotics) samples (Supplementary Table 19). Predicted pathways related to biotin metabolism, biotin biosynthesis, glycosaminoglycan degradation, vitamin B6 metabolism, and fatty acid biosynthesis pathways were decreased according to perinatal antibiotics usage. According to Bacteroides stratified data, a large portion of the above pathways relative abundances were derived from Bacteroides in both PA (37–97%) and non-PA groups (64–99%) (Supplementary Table 20). Pathways related to sugar degradation (GLUCOSE1PMETAB-PWY), pyrimidine deoxyribonucleotide biosynthesis (PWY-6545), and vitamin biosynthesis (PWY-6891) were enriched according to perinatal antibiotics usage.

## Discussion

Delivery mode and perinatal antibiotics have been shown to influence gut microbiome composition in children^1–5,7–11^. Most microbiome studies^2,4,5,7–9,11^ have used the sequencing of the bacterial 16S marker gene but have not reported the metabolic function of gut microbiome, which may mediate biological effects on the host. Whole genome sequencing of the gut microbiome would be an ideal solution but is still not feasible for every study or dataset^14,38,39^. Here, we used the PICRUSt2^15^ bioinformatics tool to predict the functional profiles of the gut microbiome based on 16S sequencing in two prospective cohorts from our laboratory. Our study shows that both Caesarean section and perinatal antibiotics markedly influence the predicted metabolic profiles of the gut microbiome at the age of 1 year.

Earlier, in a study of 60 infants using metagenome sequencing of faecal samples, perinatal antibiotics predicted microbiome alterations, but delivery mode had no enduring effects^40^. In accordance with our results, a metagenomic study by Chu et al.^41^ found several similar changes in metabolic pathways; intrapartum antibiotic exposure enriched pathways associated with glycolysis and pyrimidine metabolism whereas pathways associated with folate and biotin metabolism were decreased. Similarly, we found that predicted pathways related to sugar degradation and pyrimidine deoxyribonucleotide biosynthesis were enriched with perinatal antibiotics exposure, while biotin biosynthesis and metabolism pathways decreased.

Perinatal events affected pathways related to biotin metabolism, vitamin metabolism, and vitamin biosynthesis in the present study. Several microbial species in gut microbiome can synthesize vitamin K2 and water-soluble B-vitamins, such as biotin^42^. Vitamin metabolism genes are found across different phyla suggesting that they have a core function in gut microbiome metabolism^42,43^. In a previous metagenomic analysis of fecal samples in four countries, some of vitamin metabolism related pathways differed between study participants with type 2 diabetes or inflammatory bowel disease^42^. Overall, the clinical relevance of gut vitamin metabolism is still poorly understood but according to the present study, it appears that perinatal events, delivery mode or perinatal antibiotics, may change the microbiome-mediated vitamin metabolism in human gut of children.

In the present study, perinatal events also influenced predicted metabolic pathways of fatty acid metabolism. Short-chain fatty acids, especially butyrate, likely play an important role in the maintenance of gut health^44^. Butyrate modulates inflammatory responses and intestinal barrier function^44^. Decreased fatty acid synthesis has been reported in inflammatory bowel disease using shotgun metagenomics of faecal samples^45^ and in asthma^46^ using mass spectrometry of faecal samples for metabolome analysis. Furthermore, changes in butyrate-producing bacteria may modulate the function of nervous systems^44,47^. In an animal model, mice treated with butyrate after a high-fat diet had reduced glucose intolerance and insulin resistance and improved cardiovascular disease related metabolic disorder^48^. Thus, the observed changes in fatty acid metabolism may associate with the long-term health of children if the observed changes in the predicted metabolic pathways of gut microbiome, observed here at the age of 1 year, persist in adolescence and adulthood.

We found that genera Bacteroides to be differentially abundant according to both delivery mode and antibiotic exposure. The mean relative abundance of Bacteroides was decreased in children born by C-section and those exposed to perinatal antibiotics. According to our results, Bacteroides is a major contributor to several predicted pathways related to biotin metabolism, biotin biosynthesis, glycosaminoglycan degradation, and fatty acid biosynthesis. These predicted pathways were found to be differentially abundant or important to machine learning models according to delivery mode and perinatal antibiotics. Bacteroides contributed 64–99% of the total copy number of these pathways found in children vaginally delivered or not exposed to perinatal antibiotics. Subsequently, as relative abundance of Bacteroides decreased when exposed to C-section or perinatal antibiotics, so did the relative abundances of these pathways.

The gut microbiome is highly redundant; many different bacteria can perform the same metabolic function^12,13^. Our results suggest that not all metabolic pathways can be replenished by redundant bacteria, as several predicted pathways linked to Bacteroides were decreased in children exposed C-section or perinatal antibiotics. Still, several predicted pathways, related to proteinogenic amino acid biosynthesis and nucleotide biosynthesis were found to be important for machine learning models according to exposure to perinatal antibiotics, but not clearly decreased or increased based on mean relative abundances. These results suggest that some metabolic pathways interact with C-section and exposure to perinatal antibiotics in a complex way. Thus, mere 16S sequencing and presenting taxonomic data is not the most optimal method for microbiome research. Whole genome sequencing would be an ideal method but is still prohibitively expensive for most research groups investigating clinical associations in large study cohorts. Furthermore, currently available WGS datasets from microbiome research groups are too small for modern “data hungry” statistical methods, such as deep learning. Several tools, such as PICRUSt2^15^, Tax4Fun2^16^ and Piphillin^17^, have been developed to overcome this issue. These tools can estimate the metabolic pathway, gene or metabolite compositions of samples based on their 16S sequences. In the present study, we used PICRUSt2 to predict functional metabolic profiles of the gut microbiome in two child cohorts. Previously, PICRUSt2 was used to investigate the gut microbiome in animal models of multiple sclerosis^49^ and the gut microbiome of Crohn’s disease^50^ but rarely in prospective paediatric cohorts investigating the influence of perinatal events on subsequent microbiome composition, its function and later health.

In the present study, we analysed the gut microbiome from samples obtained at the age of 1 year in two prospective child cohorts from our laboratory^11,18,51,52^. Our predictive machine learning models confirmed the results from previous studies that both delivery mode (C-section vs vaginal delivery) and antimicrobial exposure at birth influence the abundance of Bacteroides in the gut microbiome in children^1–4,7,10,11^. In this study, we also report the novel finding that several predicted metabolic pathways linked to amino acid biosynthesis and biotin biosynthesis were identified as important when predicting unseen validation samples across the two study cohorts. However, both cohorts still showed unique differences not found in the other cohorts when analysed independently with machine learning and a traditional approach.

Recently, the machine learning approach has become popular in translational research due to its several advantages as compared to conventional statistical analysis. For instance, machine learning has been used to predict skin cancer from images^53^, type 2 diabetes from health records^54^, circumstances of death with the microbiome^55^ and exercise response to metabolic homeostasis with the microbiome^56^. Random forest, a type of machine learning algorithm, has been used to differentiate microbiome samples with taxonomic data between C-section and vaginally delivered infants^57^. Le Goellec et al. used a variety of algorithms, including random forest and other decision-tree based algorithms, to predict antibiotic usage and delivery mode from infant gut microbiome samples^58^. Stewart et al. used random forest models to examine important operational taxonomic units in predicting the age of infants in the first 40 months of life^59^. In the present study, we report the same taxonomic differences found previously. In addition, we report the predicted functional profiles of the gut microbiome according to delivery mode and perinatal antibiotic exposure using a machine learning approach. Interestingly, transforming the 16S taxonomic data to predicted metabolic pathways using the bioinformatics tool PICRUSt2 increased model performance in both cross-cohort and inner-cohort predictions. Previously published studies showed poor generalization performance when used to predict samples in a cross-study way^24,36^. Similarly, our models were unable to predict the C-section or exposure to perinatal antibiotics using only taxa as input. Using a tool to predict the metabolic pathway composition of a sample is a form of feature engineering, the process of extracting or augmenting the feature space to form new features, which is a commonly used technique in machine learning^60,61^. Contrary to most feature engineering methods, PICRUSt2 pulls new information from an outside source, such as MetaCyc^28^ dabase, to transform each feature to many new informative ones. This might be one of the reasons why our models had increased performance when using PICRUSt2 enhanced data. As such, our results suggest that predicted metabolic pathway composition may be more informative for host trait classification problems than taxonomic features alone.

In the present study, we found that mode of delivery and perinatal antibiotic exposure influenced several metabolic pathways of the gut microbiome, such as pathways for vitamin B metabolism and fatty acid synthesis. Short-chain fatty acids, especially butyrate, likely play an important role in the maintenance of gut health^44^. Butyrate modulates inflammatory responses and intestinal barrier function^44^. Decreased fatty acid synthesis has been reported in inflammatory bowel disease using shotgun metagenomics of faecal samples^45^ and in asthma^46^ using mass spectrometry of faecal samples for metabolome analysis. Furthermore, changes in butyrate-producing bacteria may modulate the function of nervous systems^44,47^. Earlier, in a study of 60 infants using metagenome sequencing of faecal samples, perinatal antibiotics predicted microbiome alterations, but delivery mode had no enduring effects^40^. In accordance with our results, a metagenomic study by Chu et al.^41^ found several similar changes in metabolic pathways; intrapartum antibiotic exposure enriched pathways associated with glycolysis and pyrimidine metabolism whereas pathways associated with folate and biotin metabolism were decreased.

The strength of the present study is that it demonstrates the successful use of predicted functional metabolic profiles based on 16S data in paediatric cohorts. There are many large clinical and translational paediatric cohorts with 16S data available, but metagenome sequencing has rarely been done due to constraints of cost and availability of whole genome sequencing of the gut microbiome. Metabolic pathways of gut microbiome data are more stable and more widely shared between individuals than taxonomic level data^62,63^. Thus, the approach presented in this study is likely to be more reasonable than mere taxonomic level comparisons. Furthermore, we had two prospective cohorts from the same laboratory for the present study. We analysed data with a machine learning approach, including cross-cohort comparisons. Our study approach shows possibilities how to utilize gut microbiome 16S datasets in a novel and meaningful way, including cross-study comparisons of functional profiles using machine learning.

The main strength of the study is also the main limitation of the study. We do not present actual metagenome sequencing data, derived from whole genome sequencing of gut microbiome, or metabolome data, derived from mass spectrometry of faecal samples, but rather use predicted functional profiles of the gut microbiome. The metabolic pathway genes of the gut microbiome predicted based on 16S data may not express the predicted proteins or the metabolites responsible for biological effects. We did not validate our results against metagenome sequencing or mass spectrometry, however, high-quality reports of PICRUSt^64^, PICRUSt2^15^, Piphillin^17^, and Tax4Fun2^16^ benchmark the methodology against WGS data in varying microbiomes. These studies show, that the predicted functional profiles strongly correlate to functional profiles from a WGS approach.

In conclusion, using two prospective paediatric cohorts, perinatal events, both Caesarean section and perinatal antibiotics, markedly influenced the functional profiles of the gut microbiome at the age of 1 year. The observed changes in metabolic pathways of gut microbiome may potentially influence the later health of children.

### Supplementary Information

Supplementary Figure 1.

Supplementary Tables.
