# Author Correction: Delivery mode and perinatal antibiotics influence the predicted metabolic pathways of the gut microbiome

**DOI:** 10.1038/s41598-023-47520-y

**Published:** 2023-11-23

**Authors:** Petri Vänni, Mysore V. Tejesvi, Sofia Ainonen, Marjo Renko, Katja Korpela, Jarmo Salo, Niko Paalanne, Terhi Tapiainen

**Affiliations:** 1 PEDEGO (Pediatrics, Dermatology, Gynecology, Obstetrics) Research Unit and Medical Research Center Oulu, University of Oulu, P.O. Box 5000, 90014 Oulu, Finland; 2 Ecology and Genetics, Faculty of Science, University of Oulu, Oulu, Finland; 3 Department of Paediatrics, University of Eastern Finland and Kuopio University Hospital, Kuopio, Finland; 4 Department of Pediatrics and Adolescent Medicine, Oulu University Hospital, Oulu, Finland; 5 Biocenter Oulu, University of Oulu, Oulu, Finland

Correction to: *Scientific Reports* https://doi.org/10.1038/s41598-021-97007-x, published online 01 September 2021

The original version of this Article contained errors in the in-text references in the Discussion and Methods sections.

In the Discussion section,

"Earlier, in a study of 60 infants using metagenome sequencing of faecal samples, perinatal antibiotics predicted microbiome alterations, but delivery mode had no enduring effects^54^."

now reads:

"Earlier, in a study of 60 infants using metagenome sequencing of faecal samples, perinatal antibiotics predicted microbiome alterations, but delivery mode had no enduring effects^60^."

60. Baumann-Dudenhoeffer, A. M., D’Souza, A. W., Tarr, P. I., Warner, B. B. & Dantas, G. Infant diet and maternal gestational weight gain predict early metabolic maturation of gut microbiomes. *Nat. Med.* **24**, 1822 (2018).

"In accordance with our results, a metagenomic study by Chu et al.^55^..."

now reads:

"In accordance with our results, a metagenomic study by Chu et al.^61^..."

61. Chu, D.M. *et al.* Maturation of the infant microbiome community structure and function across multiple body sites and in relation to mode of delivery. *Nat. Med.* **23**, 314–326 (2017).

In the Methods,

"Details about DNA extraction, primers, and sequencing protocol has been previously published for DM^18^ and PA^13^ data."

now reads:

"Details about DNA extraction, primers, and sequencing protocol has been previously published for DM^11^ and PA^19^ data."

11. Tapiainen, T. *et al. *Impact of intrapartum and postnatal antibiotics on the gut microbiome and emergence of antimicrobial resistance in infants. *Sci. Rep.* **9**, 10635 (2019).

19. Ainonen, S. *et al.* Antibiotics at birth and later antibiotic courses: Effects on gut microbiota. *Pediatr. Res.* 10.1038/s41390-021-01494-7 (2021).

The original Article has been corrected.

