# Supplementary material for: Delivery mode and perinatal antibiotics influence the predicted metabolic pathways of the gut microbiome
Source: Sci Rep. 2021 Sep 1;11:17483. doi: 10.1038/s41598-021-97007-x (PMC8410856; doi:10.1038/s41598-021-97007-x)
Supplement: Supplementary file 1 — Supplementary Figure 1. [file 41598_2021_97007_MOESM1_ESM.docx]

**
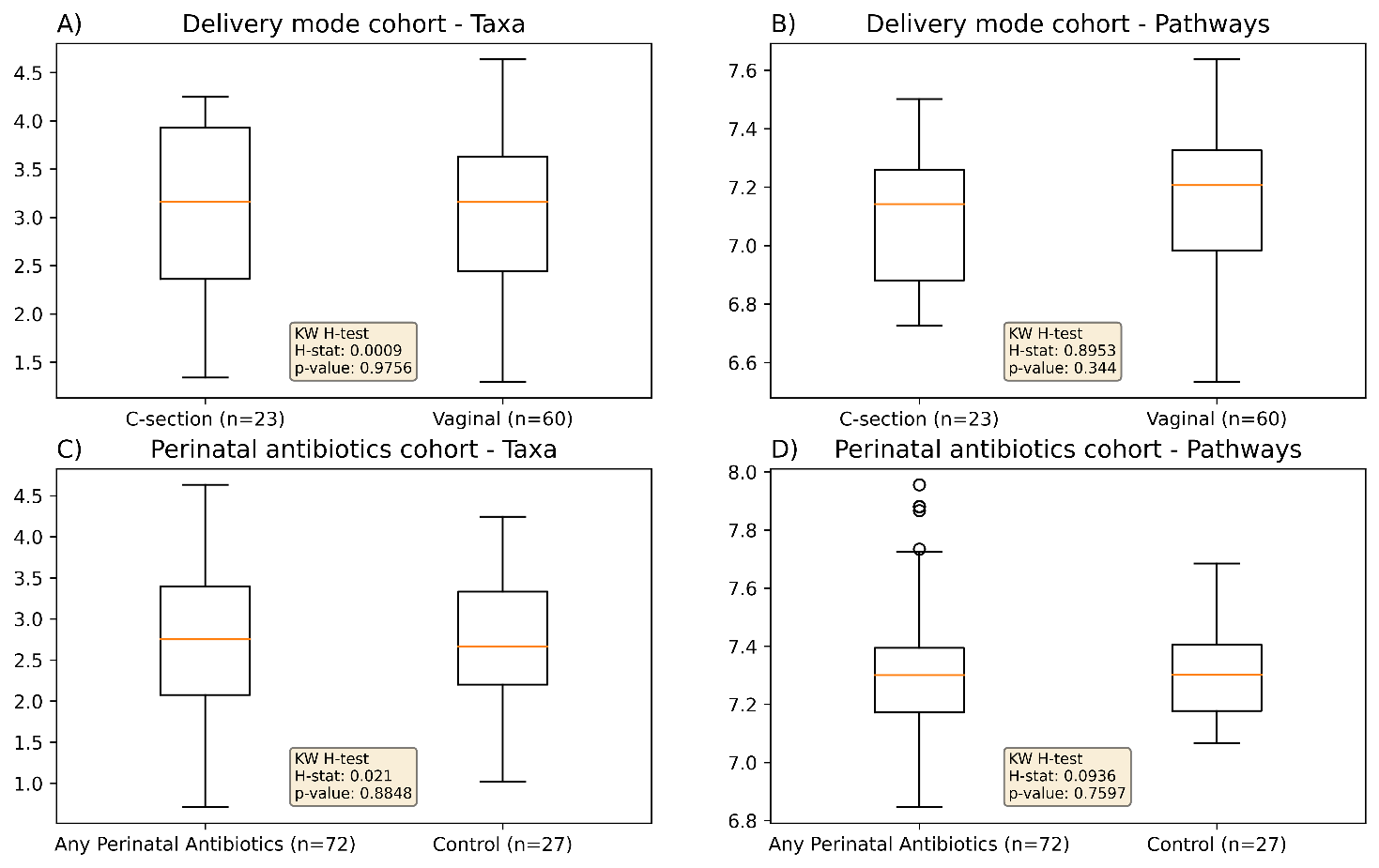
**

**Supplementary Fig 1. Within sample diversity analysis using Shannon index metric.** Rarefied taxa (A and C) and pathway data (B and D) were used in a non-phylogenetic alpha diversity analysis. Kruskal-Wallis H-test were done to statistically test the study group differences in both cohorts.
